# Detection and Management of Giant Submandibular Gland Sialolith

**DOI:** 10.12669/pjms.38.7.5988

**Published:** 2022

**Authors:** Syed Fareed Mohsin, Mohamed Abdulcader Riyaz, Abdulmalik Ali Alqazlan

**Affiliations:** 1 Syed Fareed Mohsin, Ph.D., MSc, MFD RCSI, MFDS RCPSG, BDS. Associate Professor, Oral Pathology and Oral Medicine, Department of Oral Maxillofacial Surgery and Diagnostic Sciences, College of Dentistry, Al Rass, Qassim University, Saudi Arabia; 2 Mohamed Abdulcader Riyaz, MDS, BDS. Assistant Professor of Oral Medicine and Radiology, Department of Oral Maxillofacial Surgery and Diagnostic Sciences. College of Dentistry, Al Rass, Qassim University, Saudi Arabia; 3 Abdulmalik Ali Alqazlan, 5^th^ Year Student, College of Dentistry, Al Rass, Qassim University, Saudi Arabia

**Keywords:** Sialolithiasis, Submandibular gland, Salivary gland disorder, Sialolith

## Abstract

Sialolithiasis is a disease process involving the formation of conglomerates of calcifications in the ductal system or the parenchyma of the salivary gland. The Submandibular gland is more vulnerable to form sialoliths than the other major salivary glands due to its salivary composition and anatomic factors. The management of sialolithiasis is determined by the dimensions and position of the calculi. Here, we discuss a case of a twenty eight mm submandibular sialolith managed by an intraoral approach.

## INTRODUCTION

Sialolithiasis is the most prevalent salivary gland disorder caused by stone formation within the gland or its duct. The common symptom related to this disease is pain due to gland inflammation.^1^ Sialolithiasis is the second commonest salivary gland disorder after mumps having a peak incidence in 40-50 years, with males being twice as likely to be affected as females. More than two-thirds of salivary gland calculi are found in the submandibular gland (83%), followed by the parotid gland (10%) and the sublingual gland (7%).^2^

The reasons for common involvement are alkaline pH, calcium content, viscosity, decreased salivary flow rate and anatomic factors related to the submandibular gland.^3^ The salivary sialoliths are broadly classified as Intra-glandular and Extra-glandular. The extra-glandular type is further classified as anterior and posterior sialoliths. The location of most of the salivary glandular calculi is extra-glandular. Submandibular gland calculi are 82 percent inorganic and 18 percent inorganic material, whereas parotid gland calculi constitute 51% organic and 49% of inorganic components.^4^

Salivary calculi are of varying sizes ranges from a few millimeters to centimeters. The majority of salivary calculi are less than 10 mm in diameter; those over 15 mm in diameter and occurring seldom are referred to as giant sialoliths.^5^ Although sialolithiasis is very common, giant sialoliths with diameters greater than 1.5 cm are uncommon. As a result, there are few studies in the relevant medical literature.^6^-^8^

In the following case report, we removed unusually large sialoliths from the submandibular gland by intraoral approach.

## CASE PRESENTATION

**Fig.1:**
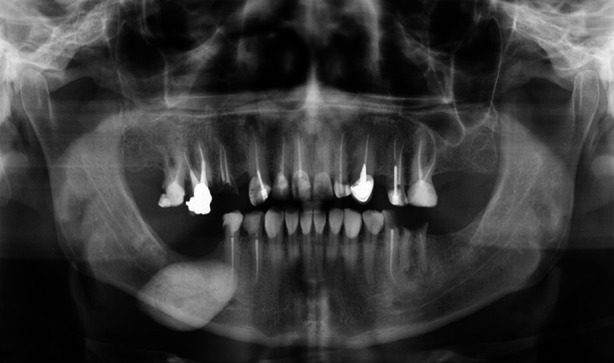
The Panoramic radiograph shows a single, roughly oval, large, well-defined radiopaque mass in the right body of the mandible.

A 47 years old male patient presented at the Qassim University Dental Hospital, Ar-Rass, Saudi Arabia, with a slowly growing lump in the right submandibular area for 15 years. The patient’s chief complaint was related to a decayed tooth. He was pre-diabetic with a history of waterpipe smoking. On bimanual examination, we noticed a moveable, firm, extensive non-tender swelling in the posterior floor of the mouth. There was no pain, discharge, or discomfort associated with the mass. In order to evaluate, a panoramic radiograph was prescribed, which revealed an enormous radiopaque calcified mass located at the right submandibular area (Fig.1).

**Fig.2:**
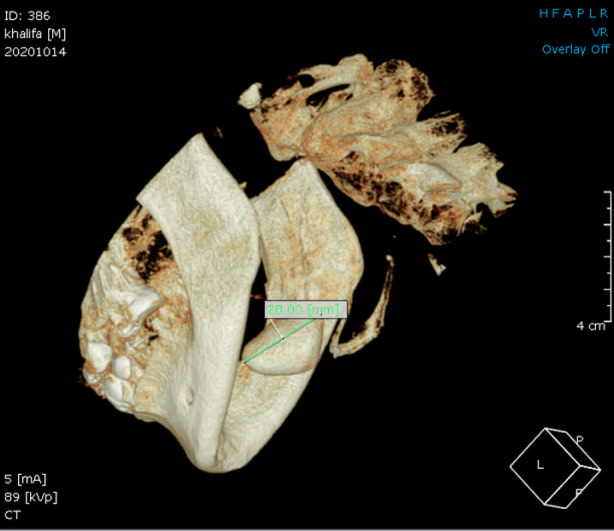
The 3D reconstructed cone beam computed tomographic (CBCT) image shows a well-defined calcified mass in the right submandibular glandular region with the maximum dimension being 28mm.

**Fig.3:**
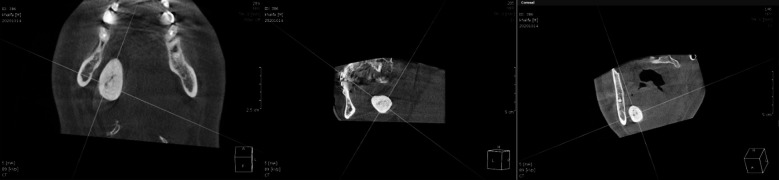
Axial, Sagittal and Coronal CBCT view reveals the concentric circular layers of calcification around the central nidus.

Furthermore, computed tomography (CBCT) was performed to confirm the diagnosis. CBCT demonstrated an oblong-shaped single giant sialolith measuring around 28 x 20 x 1.4 mm in the greatest dimension in the right submandibular gland region. The stone was well visualized in the axial, coronal, sagittal, and 3D reconstructed sections. The gray value measurement indicated that it was a well-calcified sialolith. The calcification pattern was concentric around the central nidus. Thus we made a diagnosis of submandibular sialolith. (Fig. 2 & 3).

**Fig.4:**
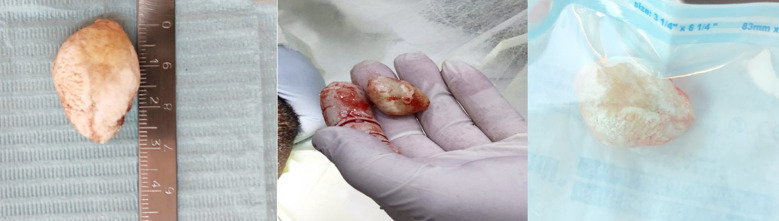
The surgically removed Sialolith with measurement.

**Fig.5:**
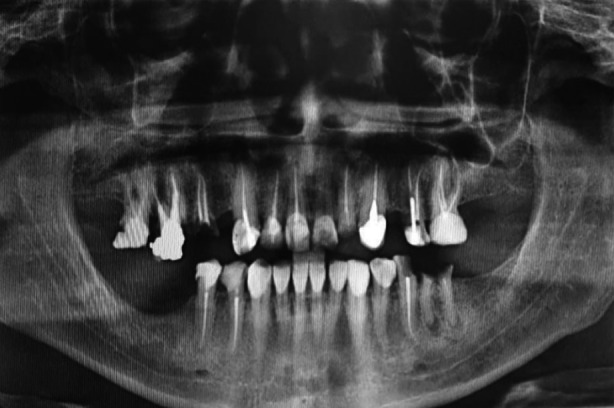
Postoperative Panoramic radiograph showed complete removal of sialolith.

On the next appointment, the sialolith was removed under local anesthesia by making an incision directly into the stone along the duct’s longitudinal axis to preserve the lingual nerve. (Fig. 4a, 4b &4c). The postoperative radiography showed complete removal of sialoliths without any complications reported by the patient. (Fig.5) The patient’s salivary gland function was assessed, which was found within the normal limit.

The biochemical analysis of the sialolith was performed using FT-IR (Fourier Transform-Infra Red) Spectroscopy which reveals the presence of calcium carbonate, calcium oxalate, and phosphate with trace amounts of protein, magnesium, and potassium.

## DISCUSSION

Sialolithiasis is one of the most frequent salivary gland pathologies of unknown etiology, having a prevalence of 1.2 % in the general population with male predominance in most of the studies reported.^2^ The submandibular salivary glands have the propensity to develop sialolithiasis due to its salivary composition, alkaline nature of its saliva, and the lengthy tortuous course of the Wharton’s duct. Calcium salt deposition is assumed to originate around an initial organic nidus of altered salivary mucins, bacteria, and desquamated epithelial cells. Other factors that may predispose a person to calculus development include salivary stagnation, salivary calcium content, inflammation, infection or physical damage of the salivary duct or gland.^9^

The patient, in this case, did not pursue dental care since he was asymptomatic, which allowed the calculus to grow in size over time. This lack of symptoms may have been due to the duct being partially blocked, allowing some saliva to be secreted and therefore causing the calculus to form without symptoms, as suggested in other cases.^10^ In the present case, even though the size of the sialolith was greater than 2.5cm, there were no signs of salivary gland dysfunction both preoperatively and postoperatively.^11^ This could be explained by the fact that the other salivary glands were functional enough to compensate for the dysfunction caused by the glandular sialolith.

Most submandibular calculi are identified on the radiograph as radiopaque formations and as radiolucent filling deficiencies on sialography. Around 20% of calculi are not radiopaque, necessitating sialography or sialendoscopy for diagnosis. Generally, salivary calculi are unilateral and do not result in dry mouth.^12^

Bodner studied various imaging methods to diagnose gigantic calculi and concluded that the panoramic radiographs and axial CT were equal in terms of their ability to estimate the calculus size before surgery.^5^ CT is quite reliable at detecting and localizing salivary calculi. Sialography should be used sparingly and should be reserved for cases of sialadenitis associated with radiolucent calculi.^13^ Since sialography is invasive, CT scans have become the primary method for identifying salivary gland calculus.^14^

The primary goal of treating sialolithiasis should be to preserve the function of the salivary glandular apparatus. The sialolith removal varies between surgeons; however, the intraoral approach is always preferred.^15^ This technique is highly straightforward and rarely results in complications. Sialadenoscopy is another minimally invasive method that can be used to treat large calculi and ductal obliteration. The CO2 laser is gaining popularity in treating sialolithiasis due to the advantages of minimum bleeding, minimal scarring, good vision, and few postoperative issues.^9^ However, removal of submandibular gland calculi is always indicated to prevent complications.

## CONCLUSION

Sialolithiasis is a salivary glands disorder, particularly in the submandibular gland. A preoperative history, clinical examination, and radiographic evaluation are essential to establish a definitive diagnosis and treatment strategy for sialolithiasis.
